# Structural and Functional Analysis of GGPPS Inhibition as a Therapeutic Mechanism for Acute Myeloid Leukemia (AML)

**DOI:** 10.21203/rs.3.rs-8370111/v1

**Published:** 2025-12-17

**Authors:** Youla Tsantrizos, Fraser Ferens, Daniel Waller, Rebecca Boutin, Hiu-Fung Lee, Marc Saba-El-Leil, Mathieu Tremblay, Tian Lai Guan, Kathryn Skorey, Michael Sebag, Arun Wiita, M Joanne Lemieux

**Affiliations:** McGill University; University of Toronto; University of California, San Francisco; McGill University; McGill University; Université de Montréal; Universite de Montreal; McGill University; McGill University; McGill University Health Centre; University of California San Francisco; Univerity of Alberta

## Abstract

Acute myeloid leukemia (AML) is a hematological malignancy with poor treatment outcomes and high mortality rates. AML progression is influenced by signalling events facilitated by small GTPases anchored to cellular membranes via post-translational modification with geranylgeranyl pyrophosphate (GGPP). The disruption of GGPP biosynthesis, and the resulting intracellular reduction of key geranylgeranylated GTPases, represents an as yet unleveraged strategy for the treatment of cancer. Here we show compound CML-07–119, a selective inhibitor of GGPP synthase (GGPPS), to display an EC_50_ potency in the nanomolar range and to induce targeted cell death in several AML cell lines, including those harbouring TP53 mutations. Antitumor efficacy *in vivo* was also observed with CML-07–119 in a mouse xenograft model engrafted with AML NOMO-1 cells, equivalent to the drug cytarabine. Bone-marrow and splenocyte cells harvested from mice treated with CML-07–119 displayed significantly higher concentration of unprenylated RAP1A as compared to the controls, demonstrating the expected biochemical outcome of in vivo GGPPS inhibition. X-ray crystallography and cryo-EM were used to determine high resolution structures of the unliganded GGPPS and the GGPPS/CML-07–119 complex. These structures revealed that the inhibitor occupies a previously proposed product inhibitory channel of the enzyme, and modulates previously unknown conformational states of GGPPS quaternary structure. This work validates GGPPS inhibition as potential novel mechanism for the treatment of AML.

## INTRODUCTION

Acute myeloid leukemia (AML) is a hematopoietic malignancy characterized by the uncontrolled proliferation of immature myeloid precursors. Of all leukemia subtypes, AML is responsible for the highest percentage of mortalities (62%), leading to a five-year survival rate of only 24% with the best current treatments. Tumor genomics play an important role on disease outcomes, and mutations or deletions of the TP53 gene (that encodes for p53 transcription factor) are known to be associated with significantly poorer drug responses in 50% of all human cancers,^,^ including AML.^,^ Although TP53 mutations are present in only 10–15% of AML cases,^,^ these mutations have been shown to increase drug resistance and are associated with particularly poor prognosis (an estimated 13% of 2-year overall survival).^,,^ Therefore, the discovery of new therapeutics for AML patients, and in particular those that harbor TP53 alterations, is of paramount importance.

**Figure 1:**
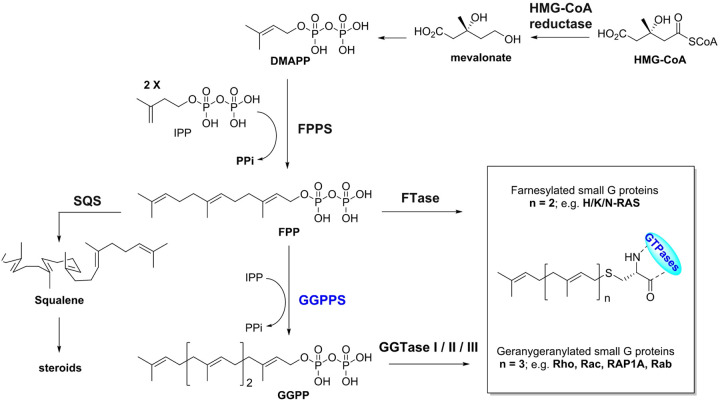
Biosynthetic pathway of isoprenoid metabolites leading to post translational prenylation of GTPases, highlighting the pivotal role of the GGPPS. Dimethylallyl pyrophosphate (DMAPP), isopentenyl pyrophosphate (IPP), squalene synthase (SQS), farnesyl transferase (FTase), geranylgeranyl transferase (GGTase).

Post-translational modification of GTP-binding proteins (GTPases) with the 20-carbon side chain of the isoprenoid metabolite geranylgeranyl pyrophosphate (Fig. 1; GGPP) allows these proteins to associate with cellular membranes and act as molecular switches that regulate cell proliferation, thus playing a significant role in cancer cell biology. Several studies have examined the role of GTPases in hematopoietic malignancies, although mainly in multiple myeloma (MM).^,,,,^ Dysregulation of the geranylgeranylated GTPase RAP1A has been shown to play a complex role in regulating tumor cell migration, invasion, and leukemogenesis. Furthermore, Rac GTPases (Rac1, 2, and 3), a subfamily of the Rho GTPases (Fig. 1), have been proposed as significant drivers in the pathophysiology of AML and have been linked to shorter overall survival.^,^ GGPP has also been reported as a pivotal metabolite for the activation of signaling by oncogenic KRAS mutants for various hematological malignancies, including AML^,^ and MM.

**Figure 2:**
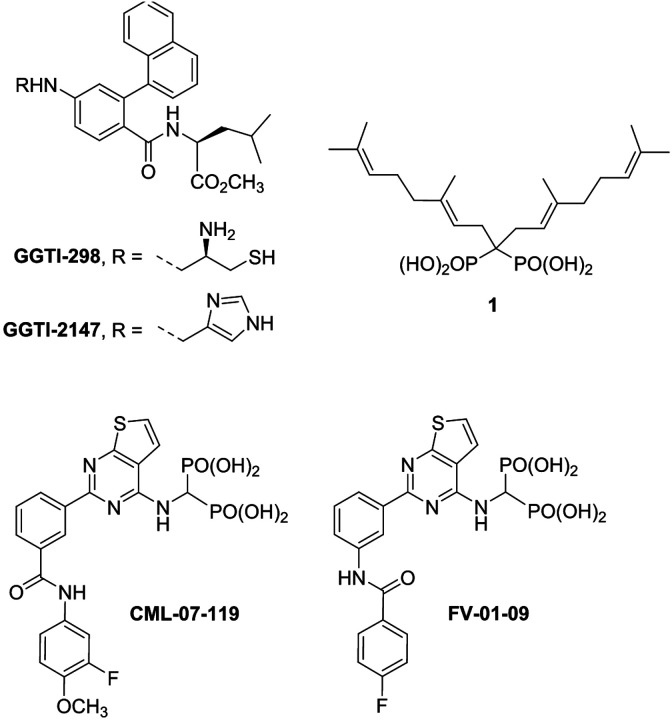
Examples of compounds that block protein geranylgeranylation in mammalian cancer cells, GGTase I inhibitors **GGTI-298** and **GGTI-2147**; GGPPS inhibitors digeranyl bisphosphonate **1**, and thienopyrimidine bisphosphonates **CML-07–119**and **FV-01–09**.

Blocking the biochemical function of prenylated GTPases (Fig. 1) can be achieved in numerous ways and has been the focus of therapeutic investigations for many years. These efforts have included blocking the transferase enzymes GGTase I/II/III, which catalyze the attachment of the 20-carbon lipidic side chain of GGPP to these GTPases, and a number of such inhibitors were evaluated, including the GGTase I inhibitors GGTI-298 and GGTI-2147 (Fig. 2). These compounds were found to be cytotoxic to AML cells only at very high and impractical (μM) concentrations. Alternative efforts (towards the treatment of AML), have focused on targeting the upstream enzymes of the mevalonate pathway, which are indirectly responsible for the biosynthesis of all the isoprenoid metabolites, including GGPP (Fig. 1). These efforts led to the evaluation of statins, targeting the 3-hydroxy-3-methylglutaryl-CoA reductase (HMG-CoA reductase), and nitrogen-containing bisphosphonates (N-BPs) that target farnesyl pyrophosphate synthase (FPPS) and block the biosynthesis FPP, which is the required substrate of GGPPS.^,^ Even farnesyltransferase (FTase) inhibitors have been explored in targeting hematopoietic malignancies; such compounds would not be expected to directly modulate geranylgeranylation of GTPases.^,,,,,,^ Although some cytotoxicity to AML cells was observed with FTase inhibitors (at high μM concentrations), this is most likely due to intracellular accumulation of FPP, which would lead to FPPS feedback inhibition and consequently, indirect inhibition of GGPPS (Fig. 1); alternative mechanisms of non-selective toxicities cannot be excluded.

To gain further insight into the role of protein prenylation in cancer, investigations into the mechanism leading to cytotoxicity in various leukemia cells by statins (including atorvastatin, cerivastatin, lovastatin,^,,^ pitavastatin, rosuvastatin,^,,^ and simvastatin,^,,^) revealed that their cytotoxic effects could be reversed by co-treatment of the cancer cells with the statin and the missing metabolite GGPP. The cytotoxic effects of N-BPs (e.g. alendronate, incadronate, and minodronate) could also be reversed with the addition of GGPP. Collectively, these studies provided evidence that the observed cytotoxicity is most likely the result of intracellular GGPP depletion, and supported the hypothesis that direct inhibition of GGPPS could be a more logical and effective mechanism for the treatment of various leukemias, including AML.

Investigations focusing specifically on selective inhibitors of the human GGPPS for the treatment of hematopoietic malignancies have so far focused mainly on multiple myeloma (MM).^14–18,^ Additionally, one report focused on acute lymphoblastic leukemia (ALL), using the digeranyl compound **1**, a selective inhibitor of GGPPS. In this study induction of apoptosis in Molt-4 and Jurkat cells was observed, with effective concentration for 50% reduction in viability (EC_50_) of 15 (4.7–48) μM and 30 (14–64) μM in Molt-4 and Jurkat cells, respectively.^53^ A second study focusing on chronic myelogenous leukemia (CML) reported similar results with structurally related GGPPS inhibitors. However, to the best our knowledge, potent and selective inhibitors of GGPPS have not been reported to induce apoptosis of AML cells, nor have been shown to induce selective intracellular engagement of GGPPS. Furthermore, the plausible variability in toxicity induced by GGPPS inhibitors to wild-type TP53 versus TP53 mutated AML cells has not been previously reported; such data may provide some insight as to the potential impact of TP53 mutations/alterations for the in vivo efficacy of GGPPS inhibitors. However, more thorough understanding would require in vivo studies for direct comparison of TP53 wt vs TP53 mutant AML.

In an effort towards the design of novel therapeutics for hematological malignancies, we investigated several structurally diverse classes of compounds that can selectively downregulate the geranylgeranylation of small GTPases by directly inhibiting the biosynthesis of GGPP.^16–18^ In this report, we provide clear evidence that inhibition of GGPPS with one of our compound, CML-07–119 (Fig. 2) leads to inhibition of AML cell viability in a small panel of AML cells that include cells with TP53 mutations. More importantly, we confirmed that our GGPPS inhibitor can also induce robust antitumor efficacy in a clinically relevant mouse model of AML, using NOMO-1 xenografts in immunodeficient NSG mice. The extent of *in vivo* efficacy observed with CML-07–119 was equivalent to that of the nucleoside drug cytarabine, which constitutes the backbone of front-line chemotherapy for newly diagnosed AML patients. Significant decreased in geranylgeranylation of RAP1A was also confirmed in both the bone marrow and splenocyte cells of the animals treated with inhibitor CML-07–119, as compared to the animals treated with vehicle, confirming the expected mechanism of action in vivo, for the observed antitumor efficacy. X-ray crystallography and cryo-EM structures of the enzyme/CML-07–119 complex revealed that this inhibitor binds to the previously reported presumptive GGPPS product inhibition channel that is potentially involved in feed-back inhibition of GGPPS. These structures represent the first high resolution GGPPS-inhibitor complexes of the wild-type recombinant human GGPPS enzyme and provide details on the interactions between a selective GGPPS inhibitor and the enzyme.

## RESULTS

### Biological Validation of GGPPS Inhibitor CML-07–119 as a Lead Molecule towards the Discovery of Novel Therapeutics for the Treatment of AML

We previously reported the identification and synthesis of thienopyrimidine-based bisphosphonate (C2-ThP-BP) inhibitors of GGPPS, such as the lead compound CML-07–119 (Fig. 2). In vitro enzyme inhibition assays indicated that CML-07–119 induces 50% inhibition in catalytic turnover of GGPPS with an IC_50_ value of ~ 27 nM, and exhibits selectivity against the most functionally related enzyme, FPPS, of approximately 50-fold.^16,17^ This inhibitor can also block the biosynthesis of GGPP in multiple myeloma (MM) cells, downregulating the post-translational geranylgeranylation of common GTPases, such as RAP1A (Fig. 1).^16,17,,,,,,^ We showed that GGPPS inhibition by CML-07–119 leads to endoplasmic reticulum (ER) stress-induced activation of the unfolded protein response and cell apoptosis.^14^ However, the induction of apoptosis in MM cells (RPMI-8226) exposed to CML-07–119 could be completely reversed upon simultaneous co-treatment of these cells with the inhibitor and geranylgeraniol (GGOH). GGOH can be taken up by cells and metabolically phosphorylated to provide the missing GGPP metabolite, which appears to be essential for cancer cell survival. These results provided strong evidence of the selective intracellular target engagement of GGPPS by inhibitor CML-07–119, and directly correlated MM cellular apoptosis with GGPPS inhibition.^16,17,18^

In the current study, a small panel of AML cell lines, carrying wild-type (MOLM-13), or mutant (Kasumi-1, NOMO-1 and THP-1) TP53 (Table 1) was selected for the initial biological screening. We are particularly interested in screening AML cells with TP53 mutations or deletions, as patients carrying these mutations have the poorest disease outcome and the highest mortality rates. Interestingly, although TP53 mutations are typically found in only ~ 10% of AML patients, after treatment with currently available therapeutics their frequency increases by approximately 5-fold; this is a clinical outcome known as treatment-related AML or t-AML.

Cell viability in the presence or absence of inhibitor CML-07–119 was evaluated using a standard MTS assay (72 hours incubation), and the concentration of inhibitor at 50% viability (EC_50_) was determined from the dose-response curve. In parallel, the toxicity induced by cytarabine was also tested in the same cell lines, as a reference of a clinically approved drug of first line chemotherapy for AML patients. The EC_50_ values in MOLM-13 (wt *TP53*) were approximately 32 nM and 5.7 nM for CML-07–119 and cytarabine, respectively (Table 1). Although the potency of both compounds was lower in all three cell lines carrying various TP53 mutations (Kasumi-1, NOMO-1, THP-1), both compounds were still fairly potent in Kasumi-1 and NOMO-1 cells, but significantly less potent or totally ineffective in blocking the proliferation of THP-1 cells at the highest concentration tested of 10 μM.

**Figure 3:**
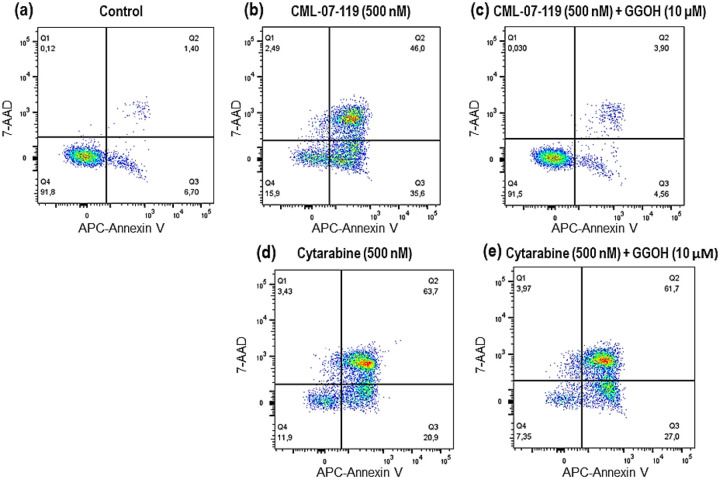
AML (MOLM-13) Cell Apoptosis Induced by GGPPS inhibitor CML-07–119 vs Cytarabine. Comparison of MOLM-13 cell apoptosis induced after 72 hours of incubation: (a) vehicle (untreated control); (b) GGPPS inhibitor (0.5 μM); (c) cell rescue by co-incubation of GGPPS inhibitor (0.5 μM) and GGOH (10 μM); (d) cytarabine (0.5 μM); (e) lack of cell rescue evidence upon co-incubation of cytarabine (0.5 μM) and GGOH (10 μM).

**Figure 4:**
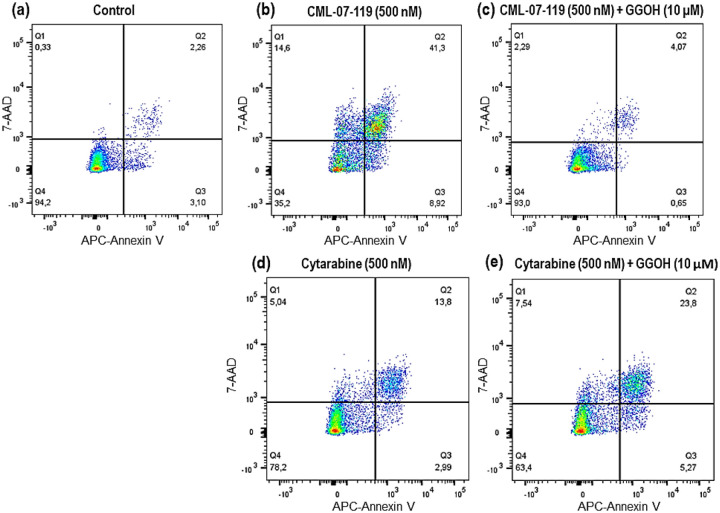
AML (NOMO-1 Luc) Cell Apoptosis Induced by GGPPS inhibitor CML-07–119 vs Cytarabine. Comparison of NOMO-1 Luc cell apoptosis induced after 72 hours of incubation: (a) vehicle (untreated control); (b) GGPPS inhibitor (0.5 μM); (c) cell rescue by co-incubation of GGPPS inhibitor (0.5 μM) and GGOH (10 μM); (d) cytarabine (0.5 μM); (e) lack of cell rescue evidence upon co-incubation of cytarabine (0.5 μM) and GGOH (10 μM).

AML cellular apoptosis was confirmed upon treatment of MOLM-13 cells with CML-07–119 by flow cytometry and complete cell rescue was observed upon co-treatment with this GGPPS inhibitor and GGOH (Fig. 3). Although cellular apoptosis was also observed with the antitumor drug cytarabine at the same concentration as CML-07–119, there was no evidence of any reversal of cytotoxicity upon co-incubation of cytarabine with GGOH, consistent with its mechanism of action being unrelated to GGPPS. These results confirm both the selective intracellular target engagement of GGPPS by inhibitor CML-07–119 in AML MOLM-13 cells, and the expected biochemical mechanism of action leading to apoptosis (Fig. 3). Identical effects were observed in NOMO-1 cells that were engineered to express firefly luciferase (Nomo-1 Luc) to permit bioluminescence tracking of tumor burden in the *in vivo* study described below (Fig. 4).

**Figure 5:**
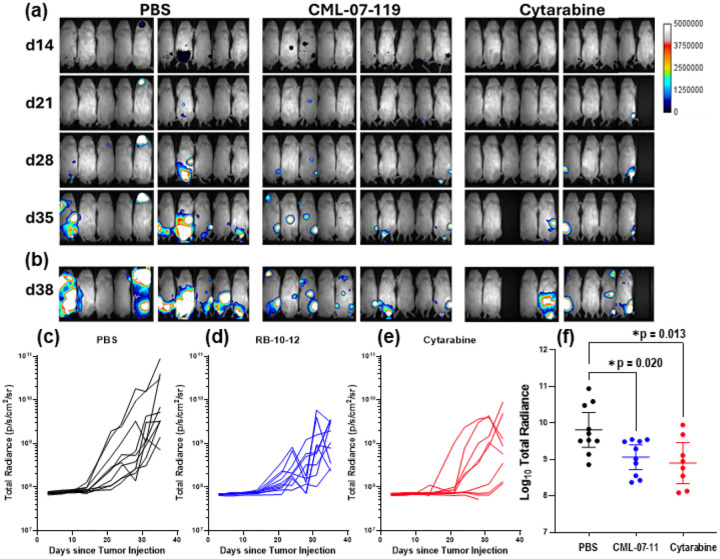
In vivo efficacy of GGPPS inhibitor CML-07–119 and cytarabine in mouse xenograft model engrafted with AML NOMO-1 cells expressing firefly luciferase (Nomo-1 Luc). (a) Examples of bioluminescence images (ventral) of male (right) and female (left) animals for each group, obtained on days 14, 21, 28 and 35 are showing tumor progression. (b) Images obtained on day 38 after 7 days without treatment. Saturation point is set to 5 × 10^7^ to avoid oversaturation of the bioluminescent signal (upper limit 5×10^6^, quantification background 5×10^3^, and output background 5×10^5^). (c) Summary of total radiance on day 38, corrected for background (d) Tumor burden analysis using total radiance (dorsal + ventral radiance values); Welch’s ANOVA demonstrated significant treatment group effect (p = 0.0116); post-hoc testing with Dunnett’s T3 demonstrated significantly lower tumor burden in RB-10–12-treated mice compared to PBS controls (p = 0.020), and in cytarabine-treated mice compared to PBS (p = 0.013).

NOMO-1 Luc cells were selected for an *in vivo* xenograft experiment, as they harbor a monoallelic TP53 frameshift mutation, yet GGPPS inhibitor CML-07–119 is reasonably potent in inhibiting their proliferation. Aliquots of these cells were administered to 8-weeks old immunodeficient NSG mice (30 males and 30 females) by intravenous injection (IV) through the tail vein. Engraftment was confirmed 7 days after injection by bioluminescence imaging, which marked day 1 of treatment initiation (Fig. 5). At that point, the mice were randomly divided into three groups (5M and 5F in each group) and dosed by intraperitoneal injection (I.P.), 3 times per week for a total of 12 doses with either vehicle (Control arm; PBS, n = 10, 5M and 5F) or 3 mg/kg of CML-07–119 as the trisodium salt dissolved in PBS (CML-07–119 arm; n = 10, 5M and 5F), or 3 mg/kg cytarabine dissolved in PBS (cytarabine arm; n = 10, 5M and 5F). Dorsal and ventral images were obtained twice per week throughout the study; examples of the images recorded at the start of each week from day 14 to 35 are shown in Fig. 5a. The weight of animals was also recorded once per week (SI Fig. 1) and no significant weight loss was observed in the control animals and the CML-07–119 treated animals. CML-07–119 treatment showed equivalent disease control to cytarabine, with none of the overt toxicities that were observed in animals treated with this nucleoside analog; a female animal in the cytarabine group was found dead on day 14, shortly after bioluminescence imaging and a male animal had to be euthanized on day 28, due to significant weight loss (20%, which is considered an ethical endpoint in our protocol). The animals in the PBS control and the CML-07–119 treated arms did not exhibit any significant signs of toxicity or death up to the end of the study.

Following the last treatment dose, a treatment-free observation period of 7 days was allowed before bioluminescence imaging was recorded again (day 38; Fig. 5b), to gain some insight on the rate of “rebound” effects of the disease, post the end of treatment. However, no strong rebound effect was observed following discontinuation of GGPPS inhibitor treatment. Overall, significant antitumor response was observed in the mice treated with GGPPS inhibitor CML-07–119, which was equivalent to that observed with cytarabine in both the male and female mice (Fig. 5c–e and 5f). Dunnett’s T3 test post-hoc statistical analysis after an ANOVA indicated significantly lower tumor burden in GGPPS inhibitor CML-07–119 treated mice compared to PBS controls (p = 0.020), as well as in cytarabine-treated mice compared to PBS (p = 0.013) (Fig. 5f).

**Figure 6:**
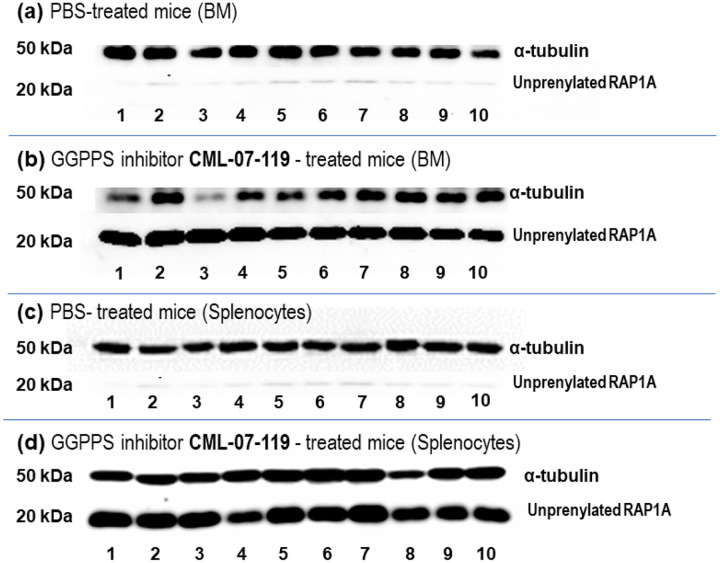
Western Blots analysis of bone marrow and splenocyte lysates isolated from NSG mice with engraftment of NOMO-1 cells expressing firefly Luciferase (Nomo-1 Luc) post treatment with PBS or GGPPS inhibitor CML-07–119 at 3 mg/kg; lanes 1–5 male ice, lanes 6–10 female mice. (a) Bone marrow lysates of mice treated with PBS (control); (b) Bone marrow lysates of mice treated with CML-07–119; (c) Splenocyte lysates of mice treated with PBS (control); (d) Splenocyte lysates of mice treated with inhibitor CML-070119. Antibodies for unprenylated RAP1A: Primary antibody Anti-RAP1A(C-17), SC1482 Lot# G1212 goat-polycolonalIgG 200μg/ml (Santa Cruz Biotechnology); Secondary antibody: Anti-goat IgG HRP HRP conjugated, SC-2354, Lot# H2119 (Santa Cruz Biotechnology). Antibodies for α-tubulin: Primary antibody: Anti-α-Tubulin, anti-mouse monoclonal, T6074–100μL, 1:1000 dilution (Sigma); Secondary antibody: Anti-mouse IgG HRP-linked, AP181P Lot#3584340 (EMD Millipore).

To gain further insight on the pharmacodynamics associated with GGPPS inhibition in the AML mouse model, at end of the study (day 38), spleens and bone marrows (BM) were harvested from animals in the control group and the CML-07–119-treated group, 16 hours following a single dose administration of either PBS or 3 mg/kg of GGPPS inhibitor, respectively. Western blots analysis of lysates from both BM cells and splenocytes confirmed target engagement and the expected downregulation of RAP1A geranylgeranylation in vivo (Fig. 6a–d).

#### Crystal and Cryo-EM Structures of CML-07–119 Bound to the Human GGPPS

**Figure 7:**
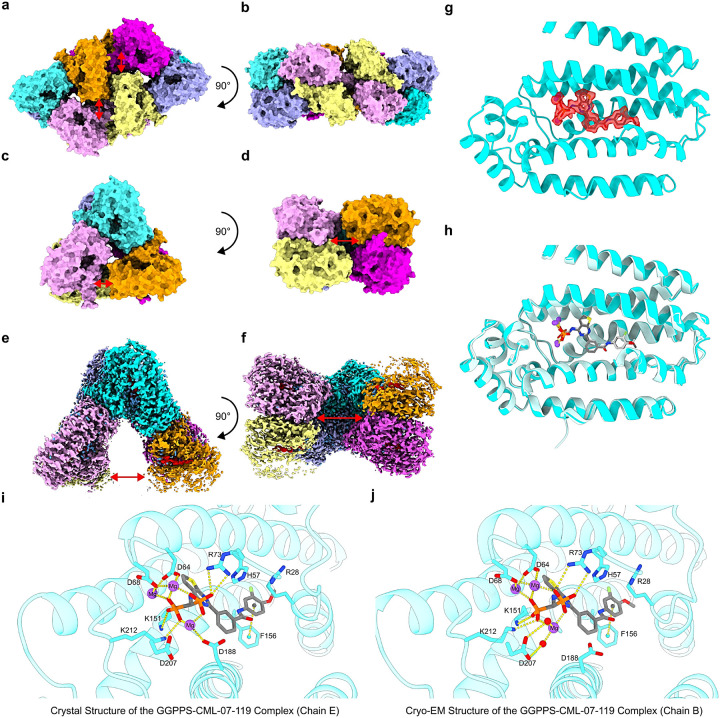
Structures of GGPPS/CML-07-119 Complexes. (a) The asymmetric unit of the GGPPS/CML-07–119 complex crystal structure; monomers of GGPPS are depicted in various colors. (c) Single hexamer of GGPPS derived from the crystal structure. (d) the GGPPS hexamer depicted in panel (b) rotated 90° around the x-axis. (e) Cryo-EM reconstruction of the GGPPS/CML-07–119 complex. (f) the reconstruction depicted in panel (e) rotated 90° around the x-axis. (g) Atomic model of a monomer of GGPPS resolved by Cryo-EM (Chain B) is represented by a ribbon diagram and the model of CML-07–119 and the Mg^2+^ ions are depicted as sticks and spheres respectively. The observed density corresponding to the inhibitor and Mg^2+^ ions is depicted as a transparent red surface. (h) The GGPPS monomer unit and CML-07–119 depicted in panel (g) (colored cyan/grey) is overlayed with the corresponding monomer unit from the crystal structure (colored pale cyan/light grey). (i) Molecular interactions between CML-07–119 and GGPPS of a representative monomer unit from the crystal structure (the monomer colored cyan in panels (a)-(c)). (j) Molecular interactions between CML-07–119 and GGPPS of the corresponding monomer unit from the Cryo-EM structure (monomer colored Cyan in panels (d) and (e)). Red arrows in panels (a)-(f) indicate the gaps observed in the GGPPS hexamers. In panels (e) and (f) the density corresponding to individual monomers of GGPPS is colored the same as the corresponding monomers from the crystal structure in panels (a)-(d), density corresponding to CML-07–119 is colored red and density corresponding to water molecules is colored blue.

To gain insight into the molecular mechanism of GGPPS inhibition, we determined both crystal and cryo-EM structures of GGPPS and the GGPPS/CML-07–119 complex. We previously reported a low-resolution co-crystal structure of inhibitor FV-01–09 (Fig. 2) bound to the GGPPS Y246D mutant (PDB code: 6C57; 3.50 Å resolution)^16^ and consequently, the precise interactions of the inhibitor with the enzyme could not be determined from this data. Here, the binding mode of CML-07–119 to the wild type GGPPS was clearly confirmed first by X-ray crystallography and subsequently by single particle cryogenic electron microscopy (Cryo-EM). The GGPPS/CML-07–119 crystal structure was solved at 2.64 Å resolution revealing an asymmetric unit containing 12 monomers of GGPPS arranged as 2 hexamers; a D3 symmetrical hexameric assembly is the expected biologically relevant form of wild-type GGPPS (Fig. 7a and 7b; Table S1; PDB: 9ZB8).^55^ Electron density corresponding to bound inhibitor molecules was observed in all monomers of GGPPS within the asymmetric unit; CML-07–119 was observed to have the same binding mode in each monomer and was located within a previously proposed product inhibition channel of the enzyme (Fig. S3a, S3b and S4).^55^

Interestingly, in each of the two hexamers within the asymmetric unit, the expected D3 symmetry of the hexamers was broken between one of the dimer-dimer interfaces with the split faces of each hexamer facing the others split face (Fig. 7a & 7b). Our purified wild-type recombinant human GGPPS was confirmed to be predominantly hexameric in solution by mass photometry (Fig. S2), consistent with previous reports that wild-type GGPPS forms a hexamer and indicating that the assembly of 2 hexamers together is a crystallographic packing artefact.^55,,^ Similar asymmetric units of the GGPPS protein was previously observed in 2 crystal structures of D188Y mutant human GGPPS (PDB: 6G31 and 6G32),^64^ however, the broken symmetry in the hexamers was presumed to be a consequence of the crystal packing artefact.^64^ Thus, we were initially concerned that this crystal form represented a non-physiologically relevant state of GGPPS, which could potentially bias our interpretation of the GGPPS-inhibitor binding interfaces. To address this concern, we pursued structural examination of the GGPPS-inhibitor complex by cryo-EM to remove the influence of crystal packing in our experimental design. Using cryo-EM we produced a reconstruction of the GGPPS/CML-07–119 complex with an overall resolution of 1.95 Å (Fig. 7e and 7f, Table S2, PDB: 9ZB9 EMDB: EMD-73979), surpassing the resolution of our crystal structure. The map of this structure contained excellent details corresponding to the bound inhibitor molecules in the presumed product inhibition channel of each of the GGPPS monomer, and the inhibitor was positioned nearly identically to our crystal structure (Fig. 7g, 7h, S3c and S3d). Importantly we found that the quaternary arrangement of GGPPS in the cryo-EM structure was very similar to our crystal structure (Fig. 4a − 4f); once again, a hexamer with broken symmetry was observed due to a large split between one of the dimer-dimer interfaces (Fig. 7c and 7d vs 7e and 7f). Taken together, the GGPPS/CML-07–119 complex was found to be comprised of a GGPPS hexamer with broken symmetry regardless of the technique used and likely represented the biologically relevant assembly of the inhibitor bound protein.

In both structures the bisphosphonate moiety of inhibitor CML-07–119 was bound to 3 Mg^+ 2^ cations that are coordinated by ionic interactions with the carboxylate side chains of D64 and D68 as previously reported for the binding of the GGPP enzymatic product (Fig. S4, 7i and 7j).^55^ Additional enzyme-inhibitor interactions include the guanidinium ion of R73, and the amine side chains of K151 and K212 forming ionic interactions with the phosphonate groups of inhibitor CML-07–119, and a π-stacking interaction between the terminal ring of the inhibitor’s side chain and the side chain of residue F156 (Fig. 7i and 7j). Although the interactions between GGPPS and CML-07–119 were found to be very similar to those observed in the crystal structure, the superior resolution of the cryo-EM reconstructions allowed for detailed modeling of the coordinated water network around the protein and inhibitor, revealing a small network of water molecules which bridged interactions between the inhibitor and the side chains of R28, H57 and R28 and the enzyme’s polypeptide backbone (Fig. S3e). Interestingly, clear density corresponding to the side chain of D188 was not observed in the cryo-EM reconstructions, indicating that this residue is not stably interacting with the nearby Mg^2+^ ion in this structure, as was observed in the crystal structure (Fig. 7i and 7j). In the cryo-EM structure the side chain of D207 was observed to form two water-mediated interactions with the Mg^2+^ ion which interacted with the side chain of D188 in the crystal structure (Fig. 7j). These water molecules were not observed in the lower resolution crystal structure, however, the side chain of D207 is in nearly the same position relative to the Mg^2+^ ion in both structures suggesting that this water bridged interaction may also be present, but that the data was not of sufficient quality to resolve these water molecules in the crystal structure (Fig. 4i and 4j). We did not concretely observe both interactions simultaneously, however, the positioning of both the water bridged interaction with D207 in the cryo-EM structure and the ionic interaction with D188 in the crystal structure would not be mutually exclusive in the octahedral geometry of this Mg^2+^ cation suggesting that there may be states of the protein where both interactions occur simultaneously. Thus, in both the crystal and cryo-EM structures of the GGPPS/CML-07–119 complex, GGPPS adopts an open hexamer conformation, and inhibitor CML-07–119 is bound to all GGPPS monomers in extremely similar orientations in both structures.

### Inhibitor CML-07–119 Prevents GGPPS from Adopting the Closed Hexamer State

**Figure 8:**
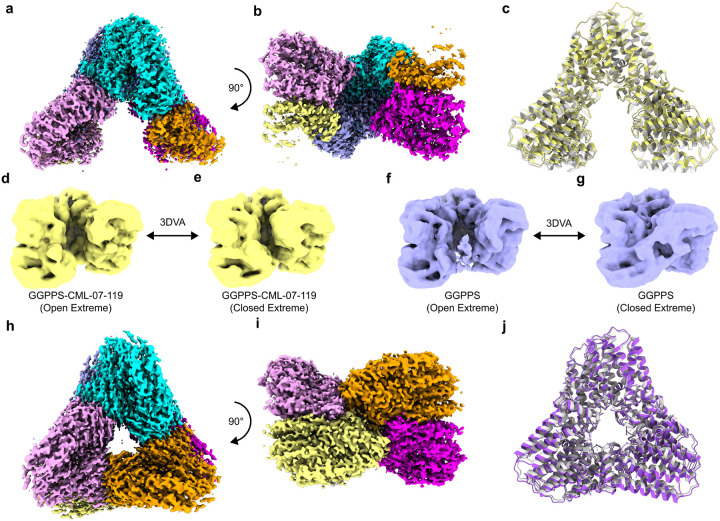
Structural evidence that CML-07–119 locks GGPPS hexamers in the open state. (a) Post-processed consensus reconstruction of the GGPPS hexamer and (b) the consensus reconstruction rotated 90° around the x-axis. (c) alignment of the GGPPS open hexamer model (yellow) with the GGPPS/CML-07–119 complex model (grey). (d) Open hexamer extreme of the 3DVA series of GGPPS/CML-07–119. (e) Farthest closed hexamer extreme of the 3DVA series of GGPPS/CML-07–119. (f) Open hexamer extreme of the 3DVA series of GGPPS lacking inhibitor. (g) Closed hexamer extreme of the 3DVA series of GGPPS lacking inhibitor. (h) Postprocessed reconstruction of GGPPS obtained from the particle subset belonging to the closed hexamer conformation identified by 3D classification and 3DVA. (i) The closed hexamer reconstruction depicted in (i) rotated 90° around the x-axis. (j) Alignment of the atomic model of the GGPPS closed hexamer conformation (purple) and the previous crystal structure of the GGPPS/GGPP complex (grey, PDB: 2Q80).

Subsequently, the possibility that the association of GGPPS with inhibitor CML-07–119 could be favoring (or stabilizing) the observed open hexamer conformation of GGPPS was considered. The cryo-EM structure of GGPPS in the absence of an inhibitor was investigated and a reconstruction with overall resolution of 2.25 Å was obtained (Fig. 8a and 8b, Table S2, PDB: 9ZBC, EMDB: EMD-73982). The unoccupied GGPPS also exhibited the same open hexamer conformation as the GGPPS/CML-07–119 complex (Fig. 8a − 8c), confirming that the binding of inhibitor CML-07–119 is not the cause of the open hexamer conformation, rather the open hexamer conformation represents a natural and likely the predominant state of the purified wild-type human GGPPS protein in solution. However, the existence of the closed D3 symmetrical GGPPS hexamer observed in prior crystal structures suggests that either there are multiple conformations that the GGPPS hexamer can adopt or that the closed D3 symmetrical hexamer is a crystallographic artefact.

A qualitative assessment of our reconstructions would suggest that there is some flexibility in the region adjacent to the opening in the hexamer structures, as the density in these monomers is blurred and fragmented, thus flexibility in this region is expected (monomers colored orange and yellow in Fig. 7e, 7f, 8a and 8b). To examine the existence of alternate conformations of the protein within our own datasets we employed 3D-Variablity Analysis (3DVA) and 3D-Classification of our particle stacks. We did not observe any reconstructions resembling the closed hexamer conformation resulting from 3D classification of the GGPPS/CML-07–119 complex dataset, however, we did observe motion of the hexamer using 3DVA which transitioned the hexamer between more open and less open extreme positions (Fig. 8d vs 8e and Movie S1). Conversely, we did observe that a reconstruction resembling the closed hexamer conformation of GGPPS was identified by 3D classification of the GGPPS sample lacking inhibitor and a motion depicting the opening and closing of the hexamer was determined using 3DVA (Fig. 8f, 8g and Movie S2). We resolved the D3 symmetric closed state of GGPPS lacking inhibitor to 2.4 Å resolution from the corresponding particle subset (Fig. 8d and 8e, Table S2, PDB: 9ZBD, EMDB: EMD-73983). The closed state of the GGPPS hexamer determined here closely aligned to the previous crystal structure of GGPPS in complex with GGPP (Fig. 8j).^55^ Collectively, these data suggest that the non-inhibited form of the protein transitions between an open and closed conformation of the hexamer while binding of inhibitor CML-07–119 to GGPPS locks the enzyme in the open hexamer conformation.

#### GGPPS Monomers Adjacent to the Opening in the Hexamer Undergo Conformational Changes

**Figure 9:**
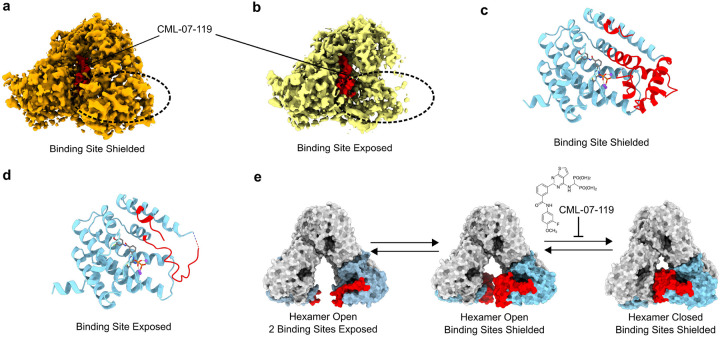
Structural Evidence of flexibility in GGPPS monomers adjacent to the hexamer opening. (a) Reconstruction of the monomer adjacent to the GGPPS hexamer opening in the GGPPS/CML-07–119 in the Binding Site Shielded state. (b) Reconstruction of the monomer adjacent to the GGPPS hexamer opening in the GGPPS/CML-07–119 in the Binding Site Exposed state. (c) Atomic model of the shielded state. (d) Atomic model of the Exposed state. (e) Model of the temporal organization of molecular motions observed in the wild-type GGPPS protein in our Cryo-EM data. In panels (a) and (b) density corresponding to CML-07–119 is colored red and the region of difference between the two reconstructions is circled with dashed lines. In panels (c) and (d) the amino acids found to be in different positions or absent in one state are colored red. In panel (e) the monomers exhibiting flexibility are colored light blue with the region of flexible amino acids colored red. Other monomers are colored light grey.

During 3DVA analysis of the GGPPS hexamer motions, we observed additional flexibility in residues 184–257 in the 2 monomers most exposed to the solvent by the opening of the hexamer of the GGPPS/CML-07–119 complex; the monomers colored orange and yellow in Fig. 7e and 7f, which are symmetry related by the C2 axis (Movie S3). Based on these observations, we aimed to compensate for domain level motion caused by the hexamer motions to better capture the local flexibility in residues 184–257. To accomplish this, we performed local refinement and 3D classification routines of the region of the particle surrounding one of these flexible monomers after symmetry expansion of the dataset. We were able to separate two distinct states of the GGPPS monomers in this position (Fig. 9a and 9b). One state corresponding to clearly structured residues 184–257, which adopted an identical fold to the other monomers of GGPPS in the hexamer, denoted as the “binding site shielded” state (Fig. 9a & 9c, Table S2, PDB: 9ZBA, EMDB: EMD-73980). In the other state density in this region is largely unobserved, indicating flexibility, however residues 185–200 and 289–296 are visibly in alternate conformations relative to the shielded state with the unresolved portion of the polypeptide chain seemingly directed away from the inhibitor binding site rather than partially shielding the binding site from the solvent (Fig. 9b and 9d, Table S2, PDB: 9ZBB, EMDB: EMD-73981), we denoted this state as the “binding site exposed” state. Notably, clear density corresponding to inhibitor CML-07–119 observed to be bound to the GGPPS monomers in both states indicated that the structural rearrangement does not lead to the dissociation of the inhibitor from the protein (Fig. 9a and 9b). Taken together, GGPPS monomers exposed to the solvent by the open hexamer state experience additional flexibility which further exposes the inhibitor (CML-07–119) and GGPPS substrate binding sites to the solvent.

## DISCUSSION

We previously reported the discovery of C2-substituted thienopyrimidine-based bisphosphonate (C2-ThP-BP) inhibitors of the human geranylgeranyl pyrophosphate synthase (GGPPS).^16,17^ These compounds can effectively block geranylgeranylation of relevant GTPases (e.g. RAP1A), induce apoptosis in blood cancer cells, such as of multiple myeloma (MM) by selective intracellular binding and inhibition of GGPPS. In the current study, we have expanded our investigations to acute myeloid leukemia (AML) and demonstrated strong in vivo antitumor efficacy in a clinically relevant mouse model with AML engraftment of NOMO-1 cells expressing firefly luciferase. Additionally, pharmacodynamic proof of the in vivo mechanism of action of the GGPPS inhibitor CML-07–119 was confirmed by western blot analysis of bone marrow and splenocyte cell lysates showing significant decrease in RAP1A geranylgeranylation as compared to the control, which are the expected consequences of GGPPS inhibition in vivo. Structural data that included X-ray crystallography and cryo-EM analysis of the GGPPS/CML-07–119 complex revealed binding of the inhibitor in the presumed product inhibition channel of the enzyme,^55^ plausibly freezing the conformational changes required for the enzyme’s optimal catalytic cycle. Although multiple binding modes of weak GGPPS inhibitors have been previously observed in the yeast enzyme,^68^ which included binding to a similar pocket, the overall interaction observed were fairly different.

Previously, 9 crystal structures of the human GGPPS had been solved, of which 2 are the wild-type protein in the D3 closed hexamer conformation (PDB codes: 2Q80,^55^ and 6R4V^64^) and 7 are GGPPS mutants assembled as the open hexamer conformation, dimers or the closed D3 hexamer conformation (Open Hexamers: 6G31 and 6G32;^64^ Dimers: 6C56 and 6C57,^16^ 9HJZ^65^ and 9CSL,; Closed Hexamer: 9HJS^65^). In addition, there are crystal structures of homologous GGPPS from other organisms such as *Saccharomyces cerevisiae* and *Plasmodium vivax*. A superposition of the active sites of the human GGPPS from the co-crystal structure with the bound catalytic product GGPP (PDB 2Q80),^55^ and the co-crystal structure of the yeast GGPPS (PDB 2E8T)^68^ with bound FsPP (a bioisostere of FPP) and IPP are shown in Fig. S3b. In the FPP binding site (commonly referred to as the allylic sub-pocket) two highly conserved aspartate-rich DDIED and DDYAN motifs chelate the 3Mg^2+^ cations, which in turn interacts with the pyrophosphate of FsPP or GGPP.^55,68^ In the IPP binding site (commonly referred to as the homoallylic sub-pocket), several positively charged residues (Arg28, His57, Arg74) mediate binding to the pyrophosphate of IPP. In the structure obtained by Kavanagh and coworkers^55^ the pyrophosphate of GGPP was observed to bind to the 3Mg^2+^ cations in the allylic site, while the geranylgeranyl side chain extended into a long hydrophobic channel consisting of valine, leucine and isoleucine residues. This channel is presumed to be a product inhibition binding site involved in feedback inhibition and is the same site occupied by our GGPPS inhibitor, CML-07–119. Thus, it is intriguing to observe the differential quaternary arrangement of GGPPS stabilized by the binding of GGPP or inhibitor CML-07–119, which are likely caused by the differences between the two molecular interfaces and GGPPS.

The displaced residues in the “binding site exposed” state include D188 and D207 which were observed to coordinate with one of three Mg^2+^ ion in our crystal structure and cryo-EM structure respectively, suggesting that monomers in the “binding site exposed” state could have reduced affinity towards the FPP substrate, or GGPP or inhibitor CML-07–119 relative to the “binding site shielded” state. Based on our collective structural data, we propose a model for the temporal arrangement of the observed molecular motions with the opening of the hexamer potentially as a requirement for the exposure of the two inhibitor binding sites (Fig. 6e). The opening of the hexamer and the destabilized portion of the GGPPS monomer structure is adjacent to the site occupied by the pyrophosphate moiety of the FPP substrate, or the GGPP catalytic product, or the bisphosphonate moiety of the CML-07–119 inhibitor in the various structures which have been discussed (Fig. 9a–9d).^55,68^ Although the function of the structural rearrangements in GGPPS observed in our data is not currently entirely clear, the localization of the observed motions is suggestive of a relationship to the catalytic cycle of the enzyme. Our current hypothesis is that the opening of the hexamer and exposure of the presumed product inhibition channel may facilitate the release of the GGPP catalytic product and re-activation of the enzyme. However, once a ligand binds to that channel, which exhibits higher affinity than GGPP, the protein may “freeze” in the open state and consequently inhibit the enzyme’s catalytic cycle. Further investigations are required to provide support for this hypothesis, which are currently in progress.

AML remains a clinical challenge to manage with the currently available therapies. Although many AML patients achieve complete remission with current chemotherapy, ~ 40% of these patients experience induction failure and do not even respond to salvage therapy nor are able to go on to allogeneic stem cell transplant. Small molecules are recently making inroads into AML care. For example, ivosidenib, a firstin-class small molecule inhibitor of isocitrate dehydrogenase-1 (IDH1), is now available for AML patients with mutated IDH1. Other small molecule therapies include the multi-target protein kinase inhibitors midostaurin, and gilteritinib, given at induction or relapse for AML with FLT3 mutations.

In order to more thoroughly evaluate the impact of GGPPS inhibition in AML, a small group of AML cell lines with wild-type (e.g. MOLM-13), or a mutated (e.g. NOMO-1, Kasumi-1 and THP-1,) TP53 genes were tested using GGPPS inhibitors CML-07–119 or cytarabine. The tumor suppressor gene TP53 is frequently mutated in human cancers, including in AML. Mutated p53 proteins may lose their tumour suppressive functions, or gain new functions that promote tumorigenesis by altering the cell cycle, apoptosis, DNA repair, inflammation and/or cell metabolism. Although the frequency of TP53 mutations in AML patients is approximately 10%, for patients with complex karyotypes, or who were previously exposed to chemotherapy for the treatment of other malignancies, it jumps to over 40%. Regardless of frequency, AML patients carrying TP53 mutations and/or deletions have very poor disease outcomes with standard chemotherapy and even allogeneic stem cell transplant. Furthermore, results using demethylating agents in combination with BCL2 inhibitors (e.g. decitabine and venetoclax) in patient with TP53 mutations have proven disappointing with short duration of responses and frequent early relapses. Our preliminary evaluation of a few AML TP53 mutant cell lines suggest that GGPPS inhibition may provide some advantages in the treatment of AML at least with some mutations, such as those found Kasumi-1 and NOMO-1. Interestingly, Kasumi-1 cells have been found to be resistant to venetoclax inhibition (reported EC_50_ value of venetoclax in Kasumi-1 cells is 5–7 μM), where as CML-07–119 exhibits nanomolar potency in this cell line (Table 1).

Drug resistance to antitumor agents is a significant challenge for cancer chemotherapy, including those aimed at treating hematological cancers. This is mainly due to tumor-induced tissue reprogramming, a mechanism largely attributable to intrinsic cellular genomic alterations induced by a given drug, underscoring the vital necessity for the discovery of new antitumor agents, with previously unexplored biochemical mechanisms of action. Inhibition of GGPPS is not yet a clinically validated mechanism for the treatment of cancer. However, our current data is encouraging and supports further efforts towards the discovery a *First-in-Class* therapeutic that selectively targets the human GGPPS and exhibits direct antitumor efficacy in AML.

## Methods

### Cell cultures:

Cell lines MOLM-13, Kasumi-1 and NOMO-1 were a generous gift from Prof. Brian Wilhelm (IRIC, University of Montreal), the THP-1 cell lines was purchased from ATCC. Cells were maintained in culture using Roswell Park Memorial Institute (RPMI) 1640 media (containing 2 mM L-glutamine, 10 mM HEPES, 1 mM sodium pyruvate, 4500 mg/L glucose, and 1500 mg/L sodium bicarbonate) supplemented with 10% FBS both from ATCC. Cultures were seeded at 5000 cells per well in a 96-well VWR^®^ Tissue Culture Plates, Non-Treated, Sterilized, Non-Pyrogenic (Lot:10861–562, VWR) in 50 mL volume. Stock solutions of compounds at 1 mM in water were serially diluted in cell media, and 50 mL of media containing a compound, or media only (control), was added to each well and incubated at 37 °C and 5% CO_2_ for 72 hours.

#### Determination of cancer cell viability.

Cell viability was determined by measuring the activity of NADPH-dependent dehydrogenases in living cells by reduction of MTS tetrazolium to formazan using the CellTiter 96-Aqueous Once Solution Cell Proliferation Assay Kit (Promega Corporation, Cat. No. G3582). Briefly, 20 mL of MTS reagent, containing a stabilized tetrazolium compound, was added to each well according to manufacturer’s instructions and incubated for 2 h at 37 °C and 5% CO_2_. The absorption at 490 nm was measured using a multimode plate reader (TECAN Infinite M200PRO) and results were normalized to give percent viability from media-only treated cells. Sigmoidal dose-response curves were fitted using non-linear regression analysis 4-parameter fit from GraphPad Prism10 (GraphPad software, San Diego, CA) and IC_50_ value at cell viability of 50% was calculated from the fit.

#### Annexin-V apoptosis assays.

To assess the ability of compounds to induce apoptosis in cultured AML cell lines, cells were seeded at a density of 7.5×10^5^/mL in RPMI-1640 medium (containing 2 mM L-glutamine, 10 mM HEPES, 1 mM sodium pyruvate, 4500 mg/L glucose, and 1500 mg/L sodium bicarbonate) supplemented with 10% FBS and the indicated concentrations of compounds. After 72 hours of treatment, apoptosis was determined by double staining with APC Annexin V (BD Biosciences, Mississauga ON) and BD Via-Probe^™^ Cell Viability Solution(7-AAD) (BD Biosciences, Mississauga ON) according to the manufacturer’s directions. Stained samples were acquired on a BD FACSCanto II instrument (BD Biosciences, Mississauga ON) and post-acquisition analyses were performed using FlowJo (V10) software. Apoptosis of the AML cells was also determined by flow cytometry by double staining cells with Allophycocyanin (APC) conjugated Annexin V and a 7-AAD vital dye for dead cells, following the manufacturer’s instructions (BD Biosciences, Mississauga ON).

#### Western Blot analysis.

AML cells were cultured in RPMI-1640 media (containing 2 mM L-glutamine, 10 mM HEPES, 1 mM sodium pyruvate, 4500 mg/L glucose, and 1500 mg/L sodium bicarbonate) supplemented with 10% FBS were maintained at 37°C in 5% CO_2_ atmosphere in the presence and absence of GGPPS inhibitor CML-07–119 or cytarabine. Geranylgeraniol (GGOH) co-treatment (at 10 μM) served as a specificity control to demonstrate bypass of GGPPS inhibitor treatment. Cells were harvested by centrifugation after the indicated treatment duration. Harvested cells were immediately washed with ice-cold PBS, centrifuged, and then resuspended in ice-cold RIPA lysis buffer (Thermo scientific^™^ cat#89900) containing 100 μM PMSF and Halt^™^ Protease Inhibitor Cocktail (Thermo scientific^™^ cat#87786). Equal amounts (20 μg) of cleared protein lysate were then separated by SDS-PAGE, transferred to PVDF membranes, and then membranes were incubated with primary antibody overnight at 4°C. The following primary antibodies were used: Anti-Rap 1A(C-17). SC1482 Lot# G1212 goat-polycolonal IgG 200μg/ml, 1:1000 dilution, from Santa Cruz Biotechnology. Anti-α-Tubulin, anti-mouse monoclonal, T6074–100μL, 1:1000 dilution, from Sigma. After extensive washing, membranes were exposed to HRP-conjugated secondary antibodies for 1 hour at room temperature. Secondary antibody: Anti-goat IgG HRP HRP conjugated. SC-2354. Lot#H2119. 200μg/0.5ml, 1:100000 dilution, from Santa Cruz Biotechnology. Anti-mouse IgG HRP-linked, AP181P Lot#3584340, 1:10000 dilution, from EMD Millipore. After further extensive washing, Clarity Western ECL Substrate, (Bio-Rad^™^ #1705061) chemiluminescence reagents were employed to visualize the remaining (bound) secondary antibodies.

#### Isolation of bone marrow cells and splenocytes

Both femur and tibia bone were crushed to release bone marrow, and the spleen tissue removed from all animals of treatment with PBS (control) and mice treated with GGPPS inhibitor CML-07–119. After the red blood cell were lysed with RBC lysis buffer, cells were resuspended in RBC lysis buffer, filtered and washed twice with PBS. Cell pellet of BM and splenocytes (which were dissociated using a mesh to obtain a single-cell suspension) were flash frozen in liquid nitrogen and kept at −80 °C until used for wester blot analysis.

### RAP1A Prenylation analysis:

MOLM-13 and NOMO-1 Luc cells were seeded in 6-well plates at density of 1×10^5^ cells/well, treated on the same day with or without compound, and incubated for 72 hours. After this incubation period, cells were harvested, and western blots were performed as previously reported.^16–18^ Similarly, lysates of bone marrow cells and splenocytes were analyzed by western blots. The following primary antibodies were used: RAP1A (C-17) (Santa Cruz) and alpha-tubulin (B-5-1-2) (EMD Millipore, Burlington, MA).

#### Mice.

Immunodeficient NSG (NOD.CgPrkdc^scid^Il2rg^tm1Wjl^/SzJ; JAX stock #005557; purchased from Jackson Laboratory) male and female mice were maintained under standard conditions at the Institute for Research in Immunology and Cancer. Mice were housed under specific pathogen-free conditions in filter-topped isolator cages with access to food and water ad libitum. Animals were handled in strict accordance with good animal practice approved by the University of Montreal Institutional Animal Care Committee in compliance with guidelines from the Canadian Council on Animal Care (CCAC).

#### In vivo anti-tumor study.

To establish engraftment of AML tumors, NSG mice (8-week-old, NOD.CgPrkdc^scid^Il2rg^tm1Wjl^/SzJ; 30 males and 30 females, purchased from Jackson Laboratory^75^) received 1.0×10^6^ NOMO-1 cells expressing the firefly luciferase gene (Nomo-1 Luc) in a volume of 200 μl PBS, administered intravenously through tail vein. Seven days later, mice were randomly divided into three groups and dosed by intraperitoneal injection (I.P.), 3 times per week for a total of 12 doses with either vehicle control (PBS, n = 10, 5 male and 5 female) or 3 mg/kg of inhibitor CML-07–119 dissolved in PBS (n = 10, 5 male and 5 female) as the trisodium salt, or 3 mg/kg cytarabine dissolved in PBS (n = 10, 5 male and 5 female). Bioluminescent imaging was performed twice a week throughout the study with the injection of 150 mg/kg of fresh sterile D-Luciferin (MediLumine). Dorsal and ventral images were obtained 15 min after the intraperitoneal injections of D-Luciferin using the LabeoTech OiS300 In Vivo Imaging System (Labeo Technologies Inc.). Signal normalization and analysis was done automatically for all time points using ImageJ (version 2.1.0/1.53d) macros and expressed in radiance (photons●s^− 1^●sr^− 1^●cm^− 2^) integrated density (Area●mean intensity). Body weights were recorded once per week and animals were observed daily for any signs of overt toxicity, such as significant weight loss, decreased mobility, or morbidity in agreement with determined ethical endpoints. Due to background instrument fluctuation of bioluminescence, the total dorsal and ventral radiance values were corrected for background and data from day 17 was removed from the plots due to the very large background variability.

### Protein Purification

GGPPS was recombinantly expressed and purified in E. coli (BL21 DE3) with an N-terminal His_6_ tag. Cells were grown in LB media at 37°C with shaking to an OD of 0.6 and were induced with the addition of IPTG to a final concentration of 0.5 mM and expression continued at 16°C overnight. Cells were harvested and lysed in 50 mM HEPES, 500 mM NaCl, 5mM imidazole, 0.5 mM TCEP, 5% glycerol, pH 7.5 in a C3 Emulsiflex (Avestin). Cell lysate was clarified via centrifugation and GGPS1 was purified via Ni-NTA chromatography with a final elution of the protein from the beads using buffer composed identically to the lysis buffer except containing 250 mM imidazole. Ni-NTA Purified GGPS1 was dialyzed into 50 mM HEPES, 500mM NaCl, 0.5mM TCEP, 5% Glycerol pH 7.5 and incubated with TEV protease overnight at 4°C to remove the His_6_ tag. Cleaved GGPPS was passed through a Ni-NTA column to remove un-cleaved protein and free tags and then was further purified using a Superdex 200 column equilibrated with 10 mM HEPES, 100 mM NaCl, 5 mM MgCl_2_, 0.5 mM TCEP, pH 7.5. Eluted GGPPS Fractions were pooled and concentrated to 62 mg/ml, aliquots were snap frozen in liquid nitrogen and stored at −80°C until needed.

### Protein crystallization and data acquisition

Inhibitor CML-07–119 dissolved in aqueous solution was added to GGPPS to a final molar ratio of 1.2:1 (Inhibitor CML-07–119:GGPPS) and the resulting solution was diluted to a GGPPS concentration of 35 mg/ml with 10 mM HEPES, 100 mM NaCl, 5 mM MgCl_2_, 0.5 mM TCEP, pH 7.5. GGPPS/CML07–119 was crystalized using the sitting drop method with a 1:1 ratio of protein solution to mother liquor. Best crystals grew in the condition: 100 mM Tris-HCl pH 8.0, 42% 2-Methyl-2,4-pentanediol (MPD). The crystals were looped and snap frozen in liquid nitrogen without the addition of additional cryoprotectant due to the high concentration of MPD in the crystallization solution. Diffraction data were collected at beamline 12 − 1 and 12 − 2 at the Stanford Synchrotron Radiation Lightsource and data were collected with the X-ray wavelength set to 0.97946 Å and at liquid nitrogen temperature.

### Cryo-EM sample preparation and data acquisition

GGPPS was incubated with inhibitor CML-07–119 as described above in the protein crystallography section or inhibitor was omitted for the sample lacking inhibitor. The resulting solutions were diluted to a final GGPPS concentration of 3.5 mg/ml in 10 mM HEPES, 100 mM NaCl, 5 mM MgCl_2_, 0.5 mM TCEP, pH 7.5. 3 μl of sample was applied to Cu 200 mesh R2/1 holey-carbon grids (Quantifoil) and were blotted and vitrified in liquid ethane using a Vitribot (Thermo Fisher) with the chamber set to 18°C and 100% humidity. Data were collected at the Pacific Northwest Cryo-EM Center on a Titan Krios (Thermo Fisher) instrument equipped with cold-FEG (Thermo Fisher), BioQuantum energy filter (Gatan) and a K3 direct electron camera (Gatan). 50 frame movies were collected with a total dose of 50 e^−^/Å^2^ at a physical pixel size of 0.8266 Å.

### Structural Biology Data Processing and Modeling

Diffraction data were initially reduced using DIALS up to the integration step and then the STARANISO anisotropy server was used to perform anisotropic scaling and merging of the data. Further data processing and modeling was conducted using the CCP4 software package. The structure was solved via molecular replacement using Phaser with a dimeric GGPPS search model extracted from a previous structure (PDB: 2Q80).^55^ The preliminary model was re-built from the phased map using ModelCraft and alternating rounds of automated refinement and manual model building were done using Refine and Coot respectively.

Cryo-EM reconstructions were produced using the cryoSPARC software package. Briefly, movies were pre-processed using Patch-Motion Correction and Patch-CTF estimation. Initial particles from subsets of data were picked using the Blob Picker and 2D classes generated from these small particle stacks were used with the Template Picker to pick particles from entire data sets. Particle stacks were cleaned with alternating rounds of Ab-initio reconstruction and heterogeneous refinement to remove junk particles until resolution ceased to improve. Ab-initio procedures were set to generate 4 classes from random subsets of the particle stack each round to generate initial reconstructions as well as junk classes for the heterogeneous refinements. Reconstructions of final particle stacks were refined using Non-Uniform Refinement with local and global CTF refinement enabled. The refined reconstructions were applied to the Reference Based Motion Correction routine to further improve the signal to noise ratio of the particles and improve the final resolution. Analysis of different states of GGPPS in the data was done using 3D classification and 3D Variability analysis routines. For the analysis of sub regions of the reconstructions, symmetry expansion was used to sample all symmetry related positions within the masked region. For full particle variability the data was re-refined with symmetry relaxation enabled to create asymmetric consensus reconstructions prior to 3D Variability Analysis. Post-processing was done using the half map method in Resolve Cryo-EM in the Phenix software package to create sharpened and density modified maps. Initial atomic models of protein for the GGPPS + CML-07–119 complex were built using ModelAngelo and all other cryo-EM models in this work utilized all or part of this atomic model as the starting model. Alternating rounds of automated refinement and manual model building were conducted using Phenix Real Space Refine and Coot^58^ respectively. Water molecules were modeled using Phenix Douse and improperly built waters were removed manually using Coot.

## Supplementary Material

This is a list of supplementary files associated with this preprint. Click to download.

- 9ZBDEMD73983valreportfullP1.pdf
- SupplementaryMovie11.gif
- SupplementaryMovie22.gif
- SupplementaryMovie31.gif
- GGPPSInhibitioninAMLSupplementaryInformationDecember152025.docx

## Figures and Tables

**Table 1:** Viability decrease (EC_50_) induced by hGGPPS inhibitor CML-07–119 and cytarabine to AML cells, with wild-type and mutant TP53

	AML				
Cell Line	MOLM-13	Kasumi-1	NOMO-1	NOMO-1-Luc^a^	THP-1
*TP53* gene	Wild-type	*TP*53 p.R248Q GOF^b^	Mono-allelic TP53 p.C242AfsTer5^c^		TP53 p.R174LfsTer3^d^
EC_50_ Values in MTS assay					
**CML-07-119**	**32** ±2 nM	**290** ± 30 nM	**110** ± 5nM	**79** ± 4.2 nM	**> 10** μM
**Cytarabine**	**5.7** ±1 nM	**26** ± 4 nM	**500** ± 45 nM	**220** ± 12 nM	**4.5** ± 0.4 μM

EC_50_ shown are the average of n = 4 determinations, each assay run in n = 6 replicates with standard deviation of ≤ 2-fold.

a NOMO-1 cells expressing firefly luciferase gene

b Gain-of-function mutation

c Frameshift mutation introducing a stop codon (Ter) five amino acids downstream from the mutation, leading to a truncated protein with loss of p53 function

d Frameshift mutation introducing a stop codon (Ter) three amino acids downstream from the mutation, leading to a truncated protein with loss of p53 function
